# Long‐Term Follow‐Up of Superior Vena Cava–Right Atrium Spontaneous Conduction Block Line Durability Using the White‐Line Approach of Extended Early Meets‐Late Rate Tools

**DOI:** 10.1002/joa3.70161

**Published:** 2025-07-31

**Authors:** Yoshiaki Mizunuma, Masao Takahashi, Takafumi Sasaki, Koichiro Yamaoka, Hirofumi Kujiraoka, Tomoyuki Arai, Rintaro Hojo, Seiji Fukamizu

**Affiliations:** ^1^ Department of Cardiology Tokyo Metropolitan Hiroo Hospital Tokyo Japan

**Keywords:** atrial fibrillation, CARTO, catheter ablation, right atrium, superior vena cava

## Abstract

**Background:**

Superior vena cava (SVC)–right atrium (RA) spontaneous conduction block occurs in some patients. We demonstrated a novel approach for SVC isolation using visualization of the SVC–RA conduction block line as a white line with the extended early meets‐late (EEML) tool of the CARTO system. The long‐term durability of SVC isolation using white line has not been investigated.

**Methods:**

Overall, 200 patients who underwent SVC isolation as atrial fibrillation therapy or additional procedures between May 2015 and April 2024 were included. We created an activation map of sinus rhythm, adjusted the EEML settings, and confirmed the white line. In the presence of a white line, we performed SVC isolation using the white line (block group); in its absence, we conducted encircling SVC isolation (nonblock group). If additional procedures were needed at follow‐up, repeat sessions were performed to identify the treatment targets, and SVC–RA mapping was performed. SVC–RA block line durability was defined as the SVC isolated area by voltage map at the additional session, including the white line of the first session. The SVC reconduction number between the two groups was compared, and SVC–RA spontaneous block line durability was confirmed in the block group.

**Results:**

Thirty‐one of 200 patients underwent additional procedures and follow‐up SVC–RA mapping. The chronic SVC reconduction ratio did not differ significantly between the two groups. SVC–RA spontaneous block line durability was maintained in all patients in the block line group (block group 12/12 [100%]).

**Conclusion:**

SVC–RA spontaneous block visualized by using the white‐line approach of EEML tools had durability in the chronic phase.

## Graphical abstract

Spontaneous superior vena cava (SVC)–right atrium (RA) conduction block lines, visualized as white lines using the extended early meets‐late (EEML) tool in the CARTO system, were associated with durable long‐term SVC isolation, supporting the efficacy of the white‐line approach.

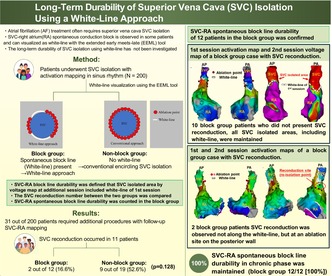

## 1 Background

The pulmonary veins are an important source of ectopic beats, initiating frequent paroxysms of atrial fibrillation [1]. These foci respond to radiofrequency ablation. Moreover, it is well known that the superior vena cava (SVC) is one of the crucial nonpulmonary vein (PV) foci of atrial fibrillation (AF) [2]. It is also known that the SVC harbors the majority of non‐PV triggers of AF. Notably, SVC isolation is feasible and safe and may be considered an adjunctive strategy to PV isolation for the ablation of AF [3]. Tanaka et al. reported that spontaneous RA–SVC conduction block can occur in some patients. They reported a novel strategy of SVC isolation using ultra‐high‐resolution mapping with SVC foci triggering AF. Patients with the spontaneous conduction block line received point‐by‐point radiofrequency (RF) applications that were delivered to connect the 2 open ends of the conduction block line, and RF applications were unnecessary along the block line to complete SVC isolation, leading to a reduced number of RF deliveries. The extended early meets‐late (EEML) feature, which indicates a white line, has been added since CARTO system Version 6. In EEML, the conduction block can be automatically visualized as a white block line, and the white line is determined based on the difference in conduction time between two adjacent mapping points. It is known that the right phrenic nerve is at risk when ablations are carried out in the SVC and the right superior pulmonary vein [4]. Inagaki et al. [5] have previously reported a new treatment method (white‐line approach) using the EEML tools of the CARTO system to visualize the RA–SVC conduction block. Nonetheless, there are no reports on the long‐term results or durability of SVC isolation using the white‐line approach.

### 1.1 Objective

This study aimed to evaluate the durability of SVC–RA spontaneous conduction block line in the chronic phase in patients who underwent SVC isolation using the white‐line approach.

## 2 Methods

**FIGURE 1:**
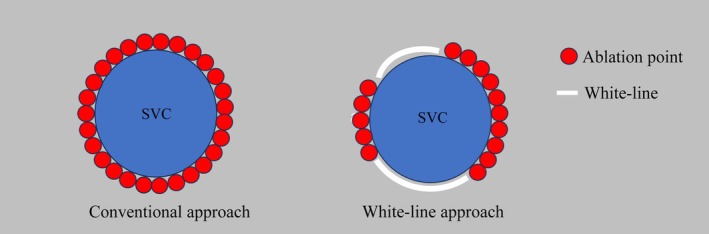
Simplified schema of conventional approach and white‐line approach of SVC isolation. This figure shows two approaches to SVC isolation. We created an activation map of sinus rhythm, adjusted the EEML settings, and confirmed the white line. When there was a white ablation line in the EEML tools, we performed a white‐line approach: SVC isolation using the white line (right figure). When there was no white line in the EEML tools, or when the white line was not used as the ablation line, we performed the conventional approach of encircling the SVC isolation (left figure). SVC, superior vena cava.

This was a single‐center retrospective study. Study participants diagnosed with paroxysmal, persistent, or long‐standing persistent AF or atrial tachycardia (AT), including patients with a history of multiple catheter ablation procedures who underwent SVC isolation between March 2015 and April 2024 at the Tokyo Metropolitan Hiroo Hospital, were enrolled. We created an activation map of sinus rhythm, adjusted the EEML settings, and confirmed the white line. When a white line was observed in the ablation line of the EEML tools, we performed a white‐line approach using (block group). When there was no white line in the EEML tools, or when the white line was not used as the ablation line, we performed a conventional approach encircling the SVC isolation (nonblock group). Figure 1 displays the conventional and white‐line schemes.

If additional procedures were needed at follow‐up, repeat sessions follow up SVC–RA map was performed to identify targets for treatment, including reconduction of the SVC.

**FIGURE 2:**
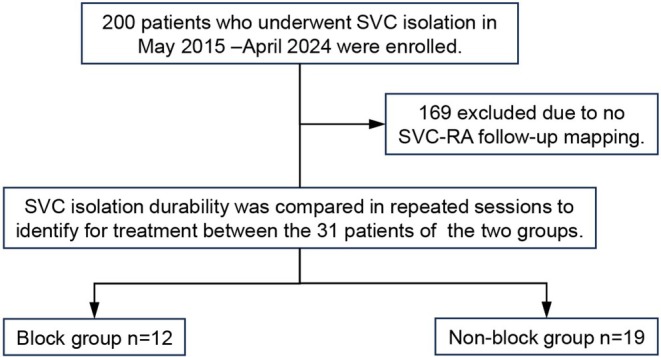
Patient flow chart. Study participants diagnosed with paroxysmal, persistent, or long‐standing persistent atrial fibrillation (AF) or atrial tachycardia, including patients with a history of multiple catheter ablation procedures who underwent SVC isolation between March 2015 and April 2024 at the Tokyo Metropolitan Hiroo Hospital, were enrolled. We created an activation map of sinus rhythm, adjusted the EEML settings, and confirmed the white line. When there was a white ablation line in the EEML tools, SVC isolation was performed using a white line (block group). When there was no white line in the EEML tools or when the white line was not used as the ablation line, we performed encircling SVC isolation (nonblock group). If additional procedures were needed at follow‐up, repeat sessions were performed to identify targets for treatment, including reconduction of the SVC. SVC isolated area durability in the block group was confirmed, and SVC isolation durability was compared between the two groups. AF, atrial fibrillation; RA, right atrium; SVC, superior vena cava.

Follow‐up RA mapping was performed, and SVC–RA block line durability was confirmed. A flowchart of the study is shown in Figure 2.

In additional catheter ablation sessions, we compared the number of SVC reconductions in both groups and confirmed the durability of the spontaneous SVC–RA block line. The study procedure complied with the principles of the Declaration of Helsinki and was approved by the Institutional Ethics Committee of the same hospital. Written informed consent was obtained from all participants. Antiarrhythmic drugs were discontinued for at least five half‐lives prior to the procedure. Oral anticoagulants were initiated at least 1 month before the procedure. The procedure was performed with the continuous intravenous administration of propofol.

### 2.1 Mapping of the RA–SVC

Activation maps were created using a mapping catheter in sinus rhythm to identify the location of the sinus node. A multipolar catheter (PentaRay catheter or Octaray or OPTRELL, Biosense Webster) was used to collect the mapping data. The electrogram acceptance criteria were as follows: (a) position stability < 2 mm, (b) local activation stability < 3 ms, and (c) maximum density. The length of the electrically activated SVC sleeve was measured from the RA–SVC junction to the highest level of the SVC sleeve at a voltage > 0.5 mV. The isolated SVC area was defined as the low‐voltage areas by voltage map.

### 2.2 EEML Setting

Setting the lower threshold (LT) in the activation map, an LT (%) was added to the CARTO 3 Version 6.0 system to highlight areas of potential conduction block called EEML. EEML divides interpolated areas with significant activation time differences. It calculates the local activation difference between adjacent points, and if this difference is higher than the percentage selected for the cycle length, a white line is drawn between those adjacent points, indicating a conduction delay or block. The system highlights any area on the map where one color interpolation differs from another. Inagaki et al. [5] reported that as a result of the retrospective evaluation of the 10 consecutive AF patients who underwent SVC isolation, the average value of the product of LAT and optimal LT/100 was 18.5 ± 3.2 ms. We assume that the conduction block exists where the local activation difference between adjacent points was more than this value. Hence, we set the first LT at 1800/LAT to optimize the visualization of spontaneous SVC–RA conduction block. We started catheter ablation by initial setting. If SVC isolation was not achieved, we adjusted the LT by increasing it by each 5% from the initial setting and visualized the optimal white line as the treatment ablation line.

### 2.3 SVC Isolation

Briefly, a circular mapping catheter (LassoNav, PentaRay, Octaray, or OPTRELL) was positioned above the RA–SVC junction. High‐output pacing was performed prior to ablation to avoid phrenic nerve injury. SVC isolation was conducted using a radiofrequency ablation catheter at 30 W, guided by the identification of the transverse nerve tag and confirmed using 20 W high‐output pacing. Radiofrequency energy was applied for up to 30 s at each point. The interlesion distances were generally kept within 4 mm to overlap CARTO VISITAG with each other and ensured lesion continuity. The treatment endpoint was defined as the disappearance of the SVC potential.

### 2.4 Definition and Confirmation of White‐Line Durability

If additional treatment was required during the postoperative follow‐up period, a repeated ablation session was performed, and the durability of SVC isolation was confirmed using SVC–RA mapping and compared between the two groups. The durability of the SVC–RA block line was confirmed in the block group. Regarding the consistency of spontaneous blocks between the initial and repeat procedures, experienced electrophysiologists carefully confirmed whether the spontaneous block lines (white lines) observed in the LAT map of the initial session for SVC isolation matched the block lines of the SVC isolated area (low voltage area) in the voltage map of the repeat session. If SVC reconduction was observed, experienced electrophysiologists confirmed where the reconduction site was. If reconduction was observed not along the white line but at an ablation site, and the low voltage area included the white line of the first session was maintained, we judged the white line to be chronically durable. This was done by individually confirming spatial consistency, based on anatomical landmarks such as the location of the earliest electrical potentials in the right atrial appendage or right atrium, to ensure that the low‐voltage areas in the repeat session corresponded to the previously identified spontaneous block lines. This supported the consistency between the two procedures.

With regard to SVC–RA isolated area durability, activation delays or bidirectional block were not evaluated in this study. If SVC reconduction occurred, we confirmed where SVC reconduction occurred, whether at the white line or at the ablation point. The LT value set was generally consistent in the first session and additional session.

### 2.5 Statistical Analysis

Data are presented as the mean ± standard deviation for continuous variables. Continuous variables were compared using the Student's *t*‐test for normally distributed variables. For categorical data, the chi‐squared test and Fisher's exact test were applied as appropriate. All reported *p* values were two‐sided, and a *p* value of < 0.05 was considered statistically significant. All statistical analyses were performed using EZR, which is used in R. More precisely, it is a modified version of the R commander designed to add statistical functions frequently used in biostatistics [6].

## 3 Results

Thirty‐one of the 200 patients underwent additional procedures. The baseline characteristics of the 31 patients are presented in Table 1. The block group had a significantly higher CHAD2 score than the nonblock group; however, there were no significant differences in the other metrics.

**TABLE 1:** Patient clinical characteristics.

	Total (*n* = 31)	Block group (*n* = 12)	Nonblock group (*n* = 19)	*p*
Age, years	62.6 ± 7.5	62.4 ± 7.8	62.7 ± 7.6	0.912
Male, *n* (%)	25 (80.6%)	11 (91.7%)	14 (73.7%)	0.363
The number of catheter ablation	2.3 ± 0.8	2.0 ± 0.9	2.5 ± 0.8	0.086
Diagnosis at first session				
Paroxysmal AF, *n* (%)	14 (45.1%)	5 (33.3%)	10 (52.6%)	0.461
Persistent AF, *n* (%)	9 (29.0%)	3 (25.0%)	6 (31.6%)	1
Long standing persistent AF, *n* (%)	7 (22.6%)	4 (33.3%)	3 (15.8%)	0.384
AT, *n* (%)	1 (3.2%)	1 (8.3%)	0	0.387
Ablation history at the time of SVC isolation (including ablation performed at the same time as SVC isolation)				
Pulmonary vein isolation, *n* (%)	30 (96.8%)	11 (91.7%)	19 (100%)	0.387
Box isolation, *n* (%)	18 (58.1%)	8 (66.7%)	10 (52.6%)	0.484
CTI ablation, *n* (%)	15 (48.4%)	4 (33.3%)	11 (57.9%)	0.387
Mitral isthmus ablation, *n* (%)	5 (16.1%)	2 (16.7%)	3 (15.8%)	1
Other ablation, *n* (%)	12 (38.7%)	4 (33.3%)	8 (42.1%)	0.717
Follow up Period between first SVC isolation session to second session, days	612.3 ± 538.4	620.8 ± 606.5	620.8 ± 434.0	0.945
CHADs score	0.9 ± 1.0	1.3 ± 1.2	0.6 ± 0.7	0.037
LAD, mm	36.9 ± 5.4	36.8 ± 5.6	37.1 ± 5.2	0.888
LAV (TTE), mL	59.0 ± 22.1	59.9 ± 18.8	57.6 ± 28.1	0.798
LAV (CT), mL	94.3 ± 24.2	91.1 ± 20.5	99.4 ± 29.7	0.437
Ejection fraction, %	61.0 ± 6.4	60.9 ± 6.9	61.2 ± 5.8	0.882

*Note:* Data are represent the mean ± SD.

Abbreviations: AF, atrial fibrillation; AT, atrial tachycardia; CT, computed tomography; CTI, cavo tricuspid isthmus; LAD, left atrium diameter; LAV, left atrium volume; TTE, transthoracic echocardiography.

Procedural characteristics of the patients in both the block and nonblock groups are shown in Table 2. The average EEML in the block group was 20.58 ± 6.26. The number of RF deliveries was lower in the block group than in the nonblock group (block group 10.58 ± 4.42 vs. nonbblock group 17.40 ± 7.68, *n* = 0.01). SVC reconduction during the additional session was observed in 11 patients (block group, 2/12 [16.7%] vs. nonblock group, 9/19 [52.6%]; *p* = 0.128). The chronic SVC reconduction ratio did not differ significantly between the two groups.

**TABLE 2:** Procedural characteristics of the patients in both the block and nonblock groups.

	Total (*n* = 31)	Block group (*n* = 12)	Nonblock group (*n* = 19)	*p*
Previous SVC isolation session				
Number of RF deliveries	14.4 ± 7.2	10.6 ± 4.4	17.4 ± 7.7	0.010
Early meets rate lower threshold, %		20.6 ± 6.3		
Sinus node dysfunction, *n* (%)	1 (3.1%)	0	1 (5.3%)	1
Phrenic nerve injury, *n* (%)	0	0	0	
Last Session (follow up SVC–RA map session)				
SVC reconduction, *n* (%)	11 (35.5%)	2 (16.7%)	9 (52.9%)	0.128
Sinus node dysfunction, *n* (%)	0	0	0	
Phrenic nerve injury, *n* (%)	1 (3.1%)	0	1 (5.3%)	1

*Note:* Procedural characteristics of the patients in both the nonblock and block groups. Number of RF deliveries are lower in block group than nonblock group. In additional catheter ablation sessions, SVC–RA mapping was performed in both groups. There was SVC reconduction in additional session observed in 11 patients (block group, 2/12 [16.7%] vs. nonblock group, 9/19 [47.4%]; *p* = 0.133). The number of SVC reconduction in the chronic phase did not differ significantly between the two groups.

Abbreviation: SVC, superior vena cava.

**FIGURE 3:**
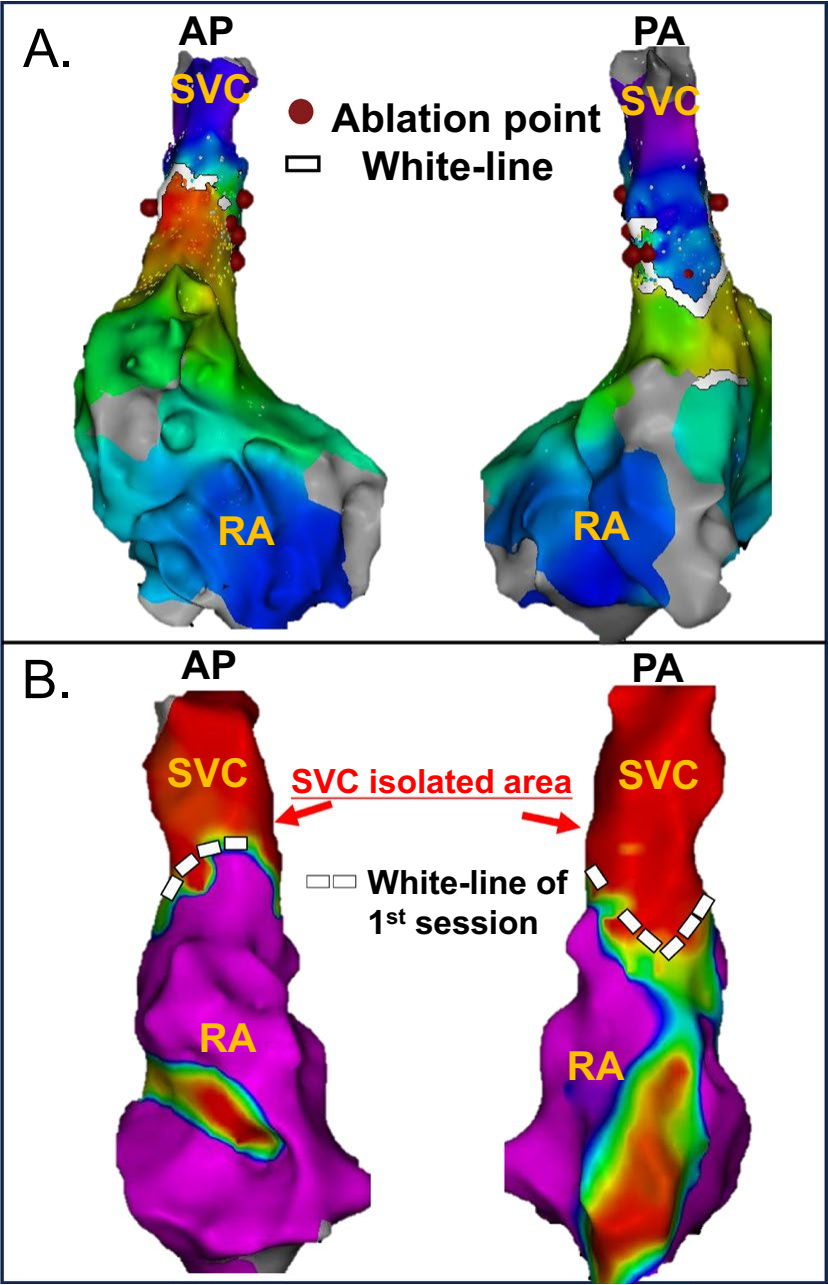
First session activation map and second session voltage map of a block group case with SVC reconduction. (A) The upper panel shows the activation map of the superior vena cava (SVC)–right atrium (RA) junction during the first session. A spontaneous SVC–RA conduction block line was identified and incorporated into the “white‐line approach.” Radiofrequency catheter ablation was performed at the septal and lateral aspects of the SVC, achieving SVC isolation without ablating along the white‐line. (B) Durable SVC isolation without evidence of reconduction was observed in the areas ablated during the initial session, despite the white‐line itself not being ablated. RA, right atrium; SVC, superior vena cava.

**FIGURE 4:**
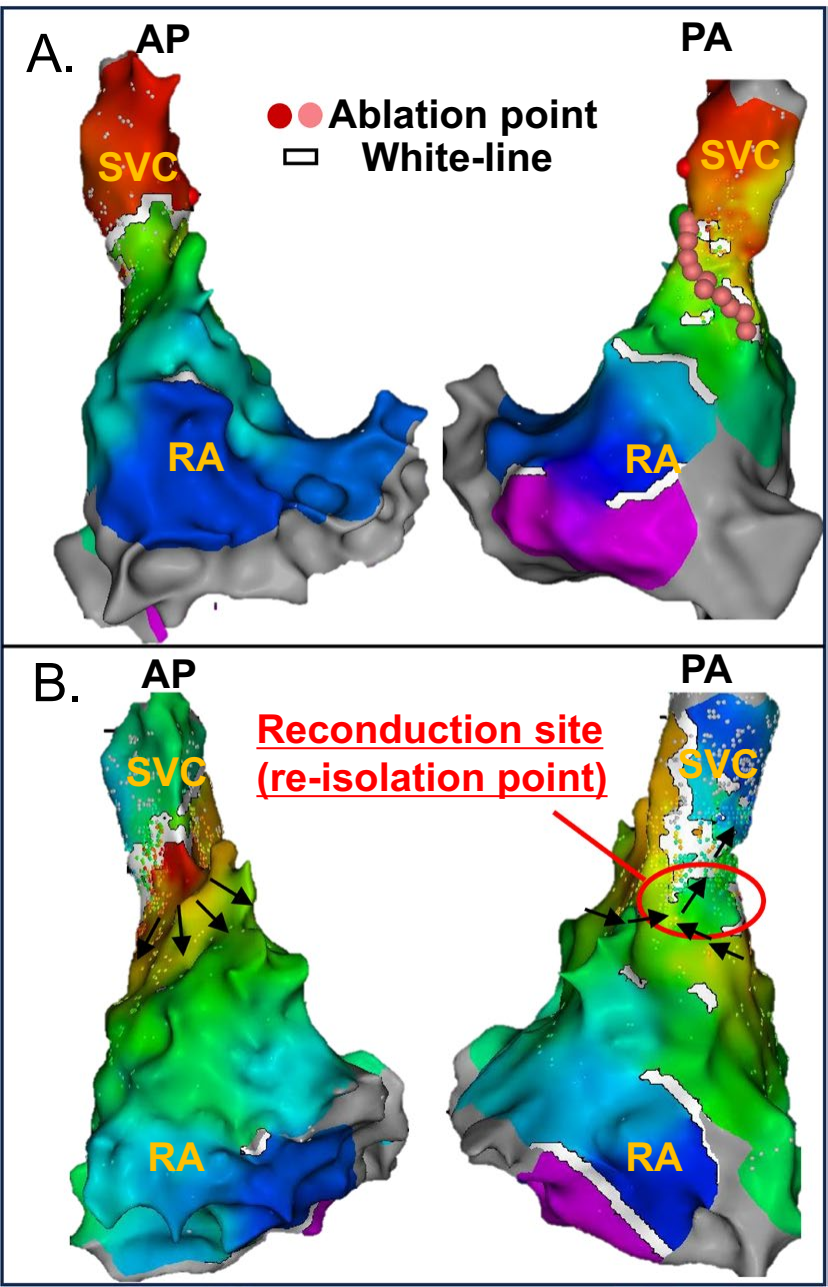
First and second session activation maps of a block group case with SVC reconduction. (A) The upper panel shows the activation map during the first session. A white line was identified from the anterior to the lateral wall of the SVC, and SVC isolation was achieved by an oblique linear radiofrequency application (pink.tag) from the septal to the posterior wall, without ablating along the white line. (B) The lower panel shows the activation map of the SVC–RA region during the second session. SVC reconduction was observed not along the white line, but at an ablation site on the posterior wall. RA, right atrium; SVC, superior vena cava.

We evaluated the durability of chronic SVC–RA spontaneous block lines. In the 10 block group patients who did not present SVC reconduction, all SVC isolated areas, including the white‐line, were maintained. For instance, we show the SVC isolated areas of block group patients who did not present SVC reconduction. The SVC isolated area, including the white‐line durability, was confirmed (Figure 3). In both cases of SVC reconduction in the block group, reconduction occurred at the ablation site rather than at the white line. For example, in one block group patient who experienced SVC reconduction, the reconduction occurred not at the white line but at the posterior SVC ablation site (Figure 4). Based on these findings, we confirmed that the chronic SVC–RA spontaneous block line durability was maintained (block group 12/12 [100%]).

## 4 Discussion

### 4.1 Major Findings

The main finding of the current study is that the SVC–RA spontaneous block line tends to maintain durability even in the chronic phase after SVC isolation using the white line approach. The results of this study indicate that it is the first to demonstrate the chronic durability of SVC isolation using the spontaneous SVC–RA block line (white‐line approach).

The nonblock group exhibited a higher rate of SVC–RA reconnection, despite undergoing more extensive ablation. In our follow‐up cohort, the nonblock group required a greater number of ablation points at the SVC–RA junction compared to the block group. One possible explanation is that a greater number of ablation points and wider lesion sets may increase the variability in lesion quality due to inconsistent contact force, varying myocardial thickness, or differences in catheter stability across the ablation area. These factors may result in nontransmural or discontinuous lesions, predisposing the site to SVC reconduction. Thus, while more extensive ablation was applied in the nonblock group, it may have resulted in less durable isolation due to these procedural limitations.

In the block group, SVC reconduction occurred at the ablation points in patients who underwent the white‐line approach, suggesting that the scar at the ablation points may have been insufficient. Although this trend could help explain the higher rate of reconduction in the nonblock group, the difference did not reach statistical significance, likely due to the limited sample size in both groups. Further studies with larger cohorts are warranted to clarify this observation.

### 4.2 Feature of Spontaneous SVC–RA Block Line and SVC–RA Block Durability

In this section, we discuss the possible mechanisms underlying the durability of the block line. One explanation for the sustained spontaneous block line is the lack of myocardial tissue with conductive properties in the SVC. Alternatively, the block line may also represent a functional conduction block.

We first consider SVC–RA spontaneous block from embryology, anatomy, and histology of SVC–RA. Generally, the formation of the caval myocardium is suggested to be the result of differentiation of mesodermal cells into heart muscle cells, rather than due to growth/migration of existing heart muscle cells [7]. The presence of sleeves of atrial myocardium continuing over the pulmonary veins and SVC has been observed in various human hearts. Previous studies in humans disclosed that the atrial wall around the superior veins was thicker and the sphincter‐like arrangement of the fibers was generally more distinct around the superior veins [8]. It was also observed that the myocardial sleeves of the superior veins usually extended further toward the lung than did the sleeves of the inferior veins. DeSimone et al. [9] reported that postmortem assessment of 620 formalin‐fixed hearts (mean age 60 ± 23 years, 44% female) was performed. The hearts were examined for integrity of venous structures and their atrial connections. Systematic gross anatomic evaluation including measurements on myocardial extensions in these veins was performed. Macroscopic myocardial extensions into the SVC occurred in 78% with the majority being circumferentially asymmetric (61%). Myocardial sleeves extend from the RA into the SVC by up to 2–5 cm and are recognized in 76% of SVC, although the RA–SVC myocardial connection is discontinuous in most cases. Hashizume et al. [10] also reported that the vena cava close to the atrium is histologically regarded as an extension of the atrium by histological study. Tanaka et al. [11] reported that the diagonal block line may constitute a functional (electrical) border between the RA and SVC and that the block line corresponds to the superior edge of the crista terminalis based on the anatomic location. Based on these previous studies, we speculate that the discontinuous RA–SVC myocardial connection may contribute to the formation of a myocardial block line, which could be visualized as a white line. If the SVC–RA spontaneous block is due to an anatomical discontinuity of myocardial tissue, its long‐term durability could be reasonably explained.

Nevertheless, this spontaneous SVC–RA conduction block also has aspects as a functional block. In our institution, Arai et al. [12] previously reported a case of SVC isolation using the white‐line approach in which acute reconnection from the RA to the SVC was observed following isoproterenol (ISP) administration. ISP is generally used for provocation of ectopic foci in the PV or SVC areas after the isolation procedure and to confirm the exit block of their impulses to the atrial tissues. The reason why ISP‐dependent SVC reconduction is explained as follows: The effect of ISP on impulse conduction may be anticipated in the region where incomplete tissue injury caused by the ablation procedure may cause partial depolarization of the resting potentials of the cardiac cells, causing inactivation of the Na^+^ current to block impulse propagation. Application of ISP on partially depolarized cells can increase the L‐type Ca^2+^ current, which induces the activation of the Ca^2+^‐dependent excitation to resume impulse conduction [13]. Therefore, if the application of ISP restores the impulse conduction in the area of the spontaneous conduction block line, the block may be explained as functional rather than fixed. This finding suggests that the white line may represent a functional conduction block that can be overcome under certain physiological conditions. Conversely, the persistence of the conduction block in the opposite direction raises the possibility that the white line may also reflect fixed conduction delay or anatomical characteristics in some regions. Therefore, based on Arai et al.'s report, the white line visualized by the EEML tool may reflect both functional and potentially fixed components of conduction block, depending on the local tissue characteristics rather than complete anatomical discontinuity. Importantly, this reconnection occurred only in the RA–SVC direction, and no reconnection from SVC to RA was observed. Therefore, we also speculated that unidirectional transient conduction from the RA to the SVC does not necessarily indicate procedural failure or require further treatment. Based on this finding, in the present study, isoproterenol was not routinely used, and bidirectional block was not assessed in all cases. The procedural endpoint was defined as the elimination of SVC potentials, which we judged to be sufficient to confirm SVC isolation.

Further investigation using high‐resolution mapping or histological correlation would be needed to fully clarify the nature of spontaneous SVC–RA block.

### 4.3 EEML Settings

As stated, the EEML reflects the relative differences in the activation time values used for color projection and is not dependent on the actual activation values themselves. Therefore, EEML software is valid for detecting conduction slowing irrespective of the arrhythmia mechanism. The thresholds of the EEML software for accurate detection of conduction blocks need to be validated in further studies. Our study adjusted EEML thresholds calculated by TCL/1800 individually, and this adjustment may be applied for more accurate visualization of conduction block zones.

### 4.4 Limitations

The following multiple factors are limitations of this study. The number of patients included in this study was relatively small. SVC isolation was performed empirically in most patients who were included in this study; therefore, the clinical implications are unknown. The surgeon was different for each case. Multiple types of mapping catheters were used and may have influenced the quality of white line visualization in EEML maps. The OPTRELL catheter, with its panel‐type design, greater number of electrodes, and reduced interelectrode spacing, may provide denser and more uniform signal acquisition compared to spline‐based catheters such as the Pentaray or Octaray. Octaray catheters and OPTRELL mapping catheters incorporate nonconductive electrodes within the catheter shaft (TRUEref Technology). This enables accurate annotation based on clearer unipolar potentials, contributing to highly accurate mapping. These structural characteristics could enhance spatial resolution and tissue contact, potentially contributing to more robust and continuous white line formation on the EEML map. A mapping system from another company was used in part during the second session. The type, power, and settings of the ablation catheter were not completely consistent. The annotation settings differed for each case. EEML settings were different for each case or session. Another limitation is that recurrence of atrial arrhythmias, including atrial fibrillation (AF) or atrial tachycardia (AT), after the additional ablation session was not systematically assessed. Since the primary aim of this study was to evaluate the electrophysiological durability of the SVC–RA block line rather than long‐term rhythm outcomes, arrhythmia recurrence after the second procedure was not included in the study protocol.

## 5 Conclusion

SVC–RA spontaneous conduction block using the white‐line approach visualized by EEML tools had durability in the chronic phase.

## Ethics Statement

We obtained approval from the Institutional Ethics Committee of Tokyo Metropolitan Hiroo hospital.

## Consent

Written informed consent was obtained from all participants.

## Conflicts of Interest

The authors declare no conflicts of interest.

## Data Availability

The data that support the findings of this study are available from the corresponding author, Yoshiaki Mizunuma, upon reasonable request.
