# Correction to “Long‐Term Follow‐Up of Superior Vena Cava–Right Atrium Spontaneous Conduction Block Line Durability Using the White‐Line Approach of Extended Early Meets‐Late Rate Tools”

**DOI:** 10.1002/joa3.70228

**Published:** 2025-11-21

Y. Mizunuma, M. Takahashi, T. Sasaki, et al., “Long‐Term Follow‐Up of Superior Vena Cava–Right Atrium Spontaneous Conduction Block Line Durability Using the White‐Line Approach of Extended Early Meets‐Late Rate Tools,” *Journal of Arrhythmia* 41, no. 4 (2025): e70161, https://doi.org/10.1002/joa3.70161.

In the originally published article, the title incorrectly included the word “Rate.” The correct title should read: “Long‐Term Follow‐Up of Superior Vena Cava–Right Atrium Spontaneous Conduction Block Line Durability Using the White‐Line Approach of Extended Early Meets Late Tools.” Furthermore, the term “Early meets‐late” was incorrectly used in several parts of the article. Specifically, this error appears in the article title, in the Background section of the Abstract (“…visualization of the SVC–RA conduction block line as a white line with the extended early meets‐late (EEML) tool…”), and in the Background section of the main text (“The extended early meets‐late (EEML) feature, which indicates a white line…”). In all of these locations, the correct wording should be “Early meets late,” without a hyphen.

In addition, in Table 2 on page 5, the label “Early meets rate lower threshold, %” should read “Early meets late lower threshold, %.”

The authors apologize for these errors.

